# Illuminating Stakeholder Perspectives at the Intersection of Air Quality Health Risk Communication and Cardiac Rehabilitation

**DOI:** 10.3390/ijerph16193603

**Published:** 2019-09-26

**Authors:** Mary Clare Hano, Christina L. Baghdikian, Steven Prince, Elisa Lazzarino, Bryan Hubbell, Elizabeth Sams, Susan Stone, Alison Davis, Wayne E. Cascio

**Affiliations:** Environmental Protection Agency, Durham, NC 27709, USA; baghdikian.christina@epa.gov (C.L.B.); prince.steven@epa.gov (S.P.); lazzarino.elisa@epa.gov (E.L.); hubbell.bryan@epa.gov (B.H.); sams.elizabeth@epa.gov (E.S.); stone.susan@epa.gov (S.S.); davis.alison@epa.gov (A.D.); cascio.wayne@epa.gov (W.E.C.)

**Keywords:** cardiac rehabilitation, air pollution, health messaging, risk communication

## Abstract

There is ample evidence of adverse cardiovascular health outcomes associated with exposure to air pollution and cardiac rehabilitation patients are at increased risk for future adverse health events related to air quality. Risk communication and health messaging about recommended behaviors to reduce exposure to air pollution can be integrated into existing care routines and structures. How this can be achieved most appropriately and effectively is not well understood. A focus group design is used to investigate cardiovascular patient and provider experiences, attitudes and beliefs about the risks of air pollution, related health risk messaging and factors that may influence integrating that topic into patient care and communication. Three discussions were hosted, one with cardiac patients, a second with non-physician cardiac rehabilitation providers and a third with physicians who treat cardiac patients. A within-case thematic inductive analysis of each discussion is used to understand the nature of communication, logistics, guidance and overall substance of the cardiac rehabilitation educational experience. Results suggest that air pollution may be an unrecognized risk factor for cardiac patients and cardiac rehabilitation is a prime setting for communicating air pollution health risk messaging. However, to effectively integrate air quality health risk messaging into cardiac rehabilitation, it is critical to account for the existing knowledge-base and behaviors of both providers and patients.

## 1. Introduction

Exposure to poor air quality poses numerous health risks. These risks include respiratory and systemic inflammation associated with particle pollution, which is made up of small particulates in the air that can travel deep within the body when inhaled [1,2]. There is ample epidemiological evidence that demonstrates clear associations between exposure to particulate matter (PM) that is 2.5 micrometers or smaller (PM2.5) and increased risk of adverse cardiovascular health outcomes [3,4,5,6,7,8,9,10,11,12,13]. Further, individuals who have experienced a cardiovascular event are at increased risk for subsequent adverse cardiovascular health outcomes associated with exposure to air pollution [14,15]. The American Heart Association, the Million Hearts initiative and the European Society of Cardiology recently included short- and long-term exposure to PM2.5 as a risk factor for cardiovascular disease [16,17,18]. The growing evidence of cardiovascular effects associated with air pollution provides the impetus for a public health response. Following a cardiac event, cardiologists may refer a patient to cardiac rehabilitation, which is separate from clinic-based care and is led by non-physician healthcare providers in a fitness gym environment [19,20,21]. Cardiac rehabilitation presents an opportunity to reach sensitive individuals when they are beyond the acute phase of the cardiac event and are actively engaged in establishing healthy habits as part of their recovery.

Public health risk messaging is a well-established approach for promoting change in health behaviors at the individual and population levels [22,23,24,25,26,27,28,29]. Health risk communication about air pollution tends to focus on individual level behaviors that reduce exposures, including for example using an air purifier, limiting time spent outdoors, minimizing indoor contributors to air pollution and so forth [1,30,31,32,33,34]. Allen et al. [35], demonstrate the physiological health benefits of air filtration. Butz et al. [36] and Chen et al. [37] provide empirical evidence of the effectiveness of air purifiers for reducing PM and lessening health symptoms associated with exposure to PM generated from anthropogenic sources. Fisk and Chan [38,39] find economic benefits generally exceed costs of individual-level strategies to reduce exposure to unhealthy air quality, particularly for individuals at risk for greater health effects from wildfire associated PM.

There is ample guidance in the public health literature on developing health risk communication programs that leverage interpersonal, physician-based and population-based messaging [40,41,42,43]. These cyclical and iterative frameworks typically include stages of planning and assessment, strategy development, message development, implementation and evaluation [22,25]. What is less well understood is how health interventions and messaging about seemingly dissimilar topics—such as air quality and cardiovascular health—can be integrated into existing patient-centered care structures and routines. The first research question guiding the present study asks: in what ways might health messaging about the cardiovascular risks of air pollution exposure be integrated into a cardiovascular rehabilitation setting?

Drawing upon the Health Belief Model [22,44,45] and the Theory of Planned Behavior [46,47,48,49] this study also examines acceptability and reception of messaging about risk reduction strategies and recommended behaviors. Both theoretical frameworks are at the individual level of analysis and suggest whether an individual actually follows a recommended health message is determined by a variety of preceding factors including their beliefs about: personal susceptibility, severity and consequences of a condition, benefits of taking action, barriers to action, ability to successfully engage in the behavior and social norms regarding the health behavior. Adoption of a recommended behavior may be associated with factors beyond the communication approach or message. For example, concerns with noise and efficacy may influence usage of filtration devices to reduce exposures [50,51]. Brugge et al. [52] found hot indoor air temperatures during warm weather seasons and whether there were family members with degenerative health conditions or a mental disability were factors decreasing acceptability of air purifiers. This study advances this vein of research and examines knowledge, attitudes, beliefs and behaviors about air quality, cardiovascular health and recommended health behaviors in the cardiac rehabilitation setting.

## 2. Materials and Methods 

In summer 2018, we conducted a series of three facilitated discussions among cardiac rehabilitation stakeholders in the southeastern United States. Each discussion was among a different stakeholder type, including patients, non-physician cardiac rehabilitation healthcare providers and physicians (cardiologists and primary care physicians). Nine individuals from each group participated. Discussions were led by a professional facilitator, were recorded and transcribed and lasted approximately 90 min. Each participant received a folder with additional background and educational materials to take home. Patients, non-physician healthcare providers and physicians have different perspectives and experiences in the rehabilitation process, thus each focus group was guided by discussion questions unique to the roles and perspectives of the stakeholders in that group. Across all three groups, we explored awareness, attitudes and beliefs about the association between air quality and cardiovascular health. Discussion guides were developed to probe our research questions around these topics of awareness, attitudes and beliefs. The specific questions guiding our study are outlined in Table 1.

The patient group included nine individuals, over the age of 65, who participated in cardiac rehabilitation following a cardiac event within the prior 12 to 18 months. The rationale for targeting individuals over the age of 65 was to focus on the age demographic with the highest susceptibility to the adverse cardiovascular effects of air pollution. The conversation with patients included discussion of their experiences in cardiac rehabilitation with an emphasis on health education; their knowledge, attitudes and beliefs about the association between unhealthy air and cardiac health; and perspectives on portable high efficiency particulate air (HEPA) purifiers. This device was included due to the growing body of research regarding the efficacy of these devices to mitigate effects of exposure [30,37,38,39].

With both groups of medical professionals, we explored processes of communication with patients and other providers, health education approaches and perspectives on incorporating air quality information into cardiac rehabilitation. Among non-physician cardiac rehabilitation healthcare providers discussion included their roles within the cardiac care and rehabilitation process, their knowledge, attitudes and beliefs about the association between unhealthy air and cardiac health and the techniques and strategies used to encourage behavior changes related to other risks. The nine individuals in this group represented a mix of non-physician healthcare providers who work in the cardiac rehabilitation setting including nutritionists, physical therapists, nurses, nurse practitioners, social workers and exercise physiologists. The physician group included six cardiologists and three primary care physicians who spend at least 30% of their time dedicated to patients over the age of 65. Questions addressed the cardiac care process from their perspective and how it intersects with cardiac rehabilitation in terms of key individuals involved, roles and responsibilities and the timeline after a cardiac event.

The research questions and objectives call for an inductive approach to analysis [53]. The analytical process involved multi-stage inductive thematic coding to reduce the data from full transcripts of the discussion into meaningful responses to the specific research questions and themes that addressed the overarching objectives [54,55]. Here, we sought to understand and characterize participant responses and dialogue to inform future outreach and research, rather than ascertaining the extent to which the responses and dialogue fit within a pre-existing conceptual framework. The transcripts were checked for quality and accuracy and the final cleaned transcript documents were analyzed. Qualitative thematic coding was done by a three-person coding team. As part of the coding process, portions of text that were salient to each question were selected and characterized using codes or brief descriptions for the specific excerpted text. First order codes and selected quotations were further refined during subsequent coding iterations for clarity and meaning, according to research question. A triangulation approach was used to validate the extent to which categories and themes we observed in the data were applicable to this broader context [54,55]. This involved consultation with experts in cardiac care rehabilitation, as well as member checking by sharing the interim findings with members of the discussion groups. Analysis was facilitated using Microsoft Office products to organize, reduce and characterize the data. All project records, including the research design development, discussion guides and analysis are maintained for reliability [53]. This study was reviewed and approved by the Office of Human Research Ethics-Institutional Review Board at the University of North Carolina-Chapel Hill.

## 3. Results

### 3.1. Patient Perspectives 

#### 3.1.1. Health Education and Behavior Change in Cardiac Rehabilitation

Patients reported that health education focused on what happened and what health behaviors they can adopt to prevent another cardiac event. Participants emphasized the value of health risk communications coming from multiple providers, both verbally and written and through a variety of mechanisms including by phone, in person at rehabilitation and during group classes at rehabilitation. Participants reported difficulty discerning whether some written risk messaging applied to them personally such as through brochures, signage, online information and so forth. When information was delivered verbally, patients perceived this information as important to them and their individual circumstance, thus increasing its relevance to their specific, personal situation. The value and impact of tailored, personalized health risk messaging was echoed across participants multiple times throughout the focus group. However, it was not just provider to patient education; peers and family also were important sources of education for the patients in this group. Connecting with others who have gone through a cardiac event and hearing their stories was a source of information and support.

“I have difficulty reading and the doctor never told us all the details of all the results. [We] arranged for my oldest daughter to have access to my file and she was able to go through and find stuff in there that [my doctor and I] never talked about at all, but [could have] significant potential or possibilities for concerns.”

Encouragements from healthcare providers were helpful to the patients’ motivation and adoption of recommended behavior changes, but most were still perceived as difficult. Patients also shared their interest and desire to learn new information but reflected that at times it can feel overwhelming. For example, the volume of written materials that patients are provided and have access to can make it challenging to keep organized and fully understand which pieces are important and applicable to their situation.

#### 3.1.2. Patient Knowledge and Perceptions of Air Quality Health Risks 

Although some patient participants noted the connection between air quality and the pulmonary system, there was very little awareness of the connection between unhealthy air quality and the cardiovascular system. These patients reported that information on air quality was not included in any of the health risk messaging they received during their cardiac care or rehabilitation. Participants were receptive to the idea that the connection existed and was important. In the quotes below, participants share their reaction to the U.S. Environmental Protection Agency handout included in their folder of background material and available air quality information resources.

“If [the U.S. EPA] are saying that [air pollution is a health risk] then obviously I am going to pay attention. Not that I believe everything the government says, but I do take that to heart.”

“I didn’t know about the sign-up for air quality emails or AirNow.gov. I didn’t know there was such a thing. And I think it would be [helpful] because the weather on television doesn’t always talk about what the air quality is.”

Further, participants questioned the need for these resources and whether an individual could assess the quality of outdoor air just by breathing or smelling it.

“If you’re breathing, you should be able to tell whether the air is good or bad when you walk out the door. That’s common sense I would think.”

#### 3.1.3. Patient Perspectives and Questions about Air Purifiers as an Exposure Reducing Technology

Generally, participants were open to the idea of using a HEPA air purifier, which is recommended by the EPA, Centers for Disease Control and Prevention (CDC) and other organizations as a strategy to reduce exposure to air pollution [34]. During this part of the conversation, participants discussed questions and factors about the device and its operation that may influence their choice to use one in their home. Their questions covered three broad categories: (1) whether they needed it in their home; (2) whether it was effective at improving the air quality; and (3) the device operation and functioning.

Questions about the necessity of these devices revolved around how patients could tell if and when the air quality in their home needed to be purified. For example, participants wanted to know if there was a specific test (like a mold or radon test kit) or some other indicator to know whether they even needed an air purifying device. Similarly, there were questions about the device’s effectiveness at improving air quality and the extent to which that improvement is reducing adverse health impacts associated with exposure to unhealthy air.

“It would be interesting to know what the air quality in our house actually was, as opposed to what’s outside. What does my house, your house, our house, what is that air quality? Does it need improving?”

From a device usage standpoint, participants were interested in learning more about purchasing costs, operating costs, reliability of the units, where the devices are sold, the capacity for the device/room size they work in and how one is properly disposed of when or if it stops functioning or is not wanted any longer. Participants also explored factors that might influence how often, when and where they might use an air purifier, including noise and ease of operation (turning it on and off, changing filters, etc.). Factors like cost and noise were considerations rather than barriers and individual preferences and health situations influenced the weight that any single factor carried in their potential decision-making process. This sentiment is captured by a participant who remarked that he could deal with any number of factors from cost to noise if it meant some of his symptoms might be reduced.

“Are we willing to spend the money for it? If I thought it was a real benefit, then I would, it’d be no problem.”

Participants emphasized that, based on their experiences, few people know about air purifiers and the potential benefit they can provide to cardiac patients. Additionally, participants also discussed that there may be challenges in educating the medical community.

“I think you have a long road ahead of you to educate the medical people because … I can’t imagine my doctor saying anything to me about an air filter. They just don’t think that far out.”

### 3.2. Non-Physician Provider Perspectives

#### 3.2.1. Non-Physician Provider Roles, Responsibilities and Patient Interactions

Participants in the non-physician provider group are involved in cardiac rehabilitation programs, which are structured health education and physical fitness programs typically lasting 12 weeks. These are fitness gym-based programs and participants come three times per week for a minimum of one hour each time. The educational aspects include formal informational classes, one-on-one provider patient interactions and small group lessons. The objective is getting patients to exercise within their current physical capacity while building strength, endurance and healthy lifestyle habits. Interactions with physicians are typically limited and occur primarily when an issue arises with a patient’s health.

Guidance focuses on the seriousness of the cardiac event, the known risk factors, effort required for sustained behavior change in diet and exercise, as well as other aspects of the patient’s life that affect their health, such as stress reduction and practical living arrangements. Notably, strong patient-to-patient communications networks also form. These may be informal or formal, like organic friendships that grow out of the shared cardiac rehabilitation experience or structured peer support groups. A cohort that starts together may perceive themselves as belonging to the same “entering class” and sometimes prior “graduates” will share information to provide their perspective on what the experience entailed and how they progressed.

#### 3.2.2. Non-Physician Provider Awareness of Risks for Cardiac Patients from Air Pollution

This group of cardiac rehabilitation healthcare providers had some awareness of the relationship between air quality and pulmonary health but not with cardiovascular health. Primary questions were related to the physical mechanisms for how air pollution can affect the cardiovascular system. These participants also reported not receiving many patient questions about air pollution in general but had fielded questions about air quality warnings seen on the news. The general response was to make decisions about physical activity (indoors or outdoors) based on how the patient feels, for example to modify exertion if tired or wheezing.

#### 3.2.3. Non-Physician Provider Strategies for Successful Patient Behavior Change

Adherence to behavior changes like diet and exercise can be difficult to sustain. Participants mentioned that psychological challenges include depression, anxiety and fear arising from the life-changing cardiac event. These problems can be compounded by stressors arising from other areas of life, including for example financial, interpersonal or even lack of access to transportation. Strategies participants relied on to encourage sustained health behaviors include building rapport, message repetition, establishing an environment where healthy choices are the norm among peers and empowering family members to ask questions and feel like a resource for patient encouragement and support outside the program. Family members are sometimes coached to reinforce the health behaviors at home and participate in creating a plan to improve success for patient adherence to new routines and behaviors.

“We’ve got numbers for family members if we can’t get in touch with the patient. Backups to the backup…We get to know everybody. Make them all part of the process…It’s important [to have family involved]. They’ve got to come back and stick to that routine, especially after rehab[ilitation].”

Message tailoring and repetition were also strategies these participants relied on for encouraging patients in adopting healthy behaviors. This came across in both verbal health messaging shared with patients as well as visual reinforcements and signage posted in the cardiac rehabilitation environment. Additionally, all messaging and communication is designed at a fifth-grade reading level. To encourage continued efforts at change, it is important to celebrate small steps and successes. This social recognition was also a tool to establish social norms and demonstrate to patients that these behavior changes are doable and common among their peers.

“We put little stars on their charts. They have paper charts as well as we have the computer charts. We give them little stars to make note of it.”

### 3.3. Cardiologist and Primary Care Physician Perspective 

#### 3.3.1. Physician Perspective on Cardiovascular Rehabilitation

Once the immediacy or acute phase of the cardiac event has passed, much of the health messaging from both cardiologists and primary care physicians (PCPs) focuses on diet, exercise and compliance with interventions and medications. PCPs have an important role in understanding the whole patient, for example their psychological health, social environment and financial constraints. These factors may influence compliance with recommended treatments and health behavior recommendations and PCPs work to find a plan that fits within the patient’s broader circumstances, like examining medication or fitness options in the context of the patient’s budget. This broader perspective is important for continued adherence to recommended behavior changes. Patients may be overwhelmed and not aware of important questions during the cardiologist visit. PCPs can fill in once the patient is ready to process the information.

“The patient has most likely already seen the cardiologist for their first follow up so my role [as PCP] is reinforcing the messages that they’ve been getting and trying to get them to commit to the medications, the lifestyle changes and cardiac rehab[ilitation]. They will often have a lot of questions. I don’t know why but they often don’t ask the cardiologist everything that’s on their mind, so we fill in the gaps and reinforce the message that they’ve gotten already.”

Physician to patient health education is typically limited to relatively brief clinic appointments. The emphasis of that messaging changes over time, beginning with the cardiac event as a symptom of underlying disease and that the inpatient intervention (such as a stent, bypass, etc.) was a strategy to address the symptom rather than curing the underlying disease and expanding to broader health behavior changes to reduce risk of recurrence. They also provide handouts and pamphlets to supplement topics that may not have been covered in-depth during the clinic visit.

The recommendation to go to cardiac rehabilitation is essentially standard protocol following most cardiovascular events. Physician trust in rehabilitation staff is high and both cardiologists and PCPs communicated the need and importance of rehabilitation to the patient during the recovery process. Once the patient enters cardiac rehabilitation, the interaction among the non-physician healthcare providers and the cardiologist is limited to updates and health issues that may arise. However, not all patients can or will go to cardiac rehabilitation. In these instances, PCP messaging becomes increasingly important. Behavior change is incredibly difficult; even among patients who go through cardiac rehabilitation, physicians reported that observing sustained change over time is rare. The exception is patients who are younger, already exercising or those unusually motivated to do exercise and accept dietary change.

#### 3.3.2. Physician Perspectives on Integrating Air Quality Health Messaging into Cardiac Care

Physician participants in this group had limited pre-existing awareness of the connection between air pollution and cardiovascular health and the increased risk for cardiac patients compared to the general population. The link between pulmonary health and air pollution was much clearer and well understood. However, the connection with cardiovascular health was not totally foreign nor dismissed as implausible. Concerns about integrating health messaging about air pollution were related to time with patient and empirical evidence related to the risk and recommended behaviors. There were questions about the extent to which an individual could modify their exposure to air pollution, indoors or outdoors. Further, during clinic visits physicians must prioritize topics and often limit conversation to high impact messages. These participants had questions about whether the empirical evidence about air pollution and cardiac health justifies elevating the topic during a brief clinic visit. While some participants were open to the idea of including it during later stages of recovery, others believed they should focus on behaviors that have a high impact on health.

“Well I would say it should be a part of trying to take care of the patient. I’m not saying this is number one priority, but if you’re talking about risk and exposures and how to avoid something, then you have to include it.”

“It’s all about priorities. You have to prioritize what you’re discussing with your patient, because you don’t want to give them a whole bunch of information. … It’s proven that less than 50% of what you tell a patient they don’t remember as soon as they step out of your room.”

“I’d like to know the absolute risk increase. I mean, are we talking about going from 0.01% to 0.02%? That’s a doubling of risk, but it’s inconsequential to me and probably to most of my patients. So, I need to learn more about what it is, what’s the real effect, what’s the absolute risk increase and maybe studies that show whether interventions like air filters or things like that in the home, do they benefit anybody?”

#### 3.3.3. Physician Perspectives on Opportunities for Integrating Air Quality Health Risk Messaging 

Participants suggested introducing the idea after the initial event and recovery phase may be more appropriate given limited time with patients. Leveraging the clinic-based, non-physician providers as educators on the topic was also discussed as an approach to incorporating the topic into a clinic visit.

A summary of findings from each group is included in Table 2.

## 4. Discussion

We began this study with the intention of engaging in initial stages of a health risk communication framework [23,56] by collecting and analyzing insights for the purpose of developing health risk messaging. Cardiac rehabilitation is a prime opportunity to connect with individuals who are at increased risk of adverse health outcomes from exposure to air pollution. The patients are primed for learning and are motivated to engage in behaviors that may be new to them. However, across all three groups this analysis revealed little awareness of the cardiac health risks of air pollution. Air pollution health risk communication programs can consider broader audiences beyond patients, to include physicians, other healthcare providers and family members or friends of cardiac patients. 

Because healthcare providers are the primary and trusted health educators, education and outreach materials geared toward them are critical to integrating the topic into patient care. Additional resources are needed like the continuing education course, “Particle Pollution and Your Patient’s Health” [57] to close the gap between empirical evidence of the issue and provider awareness. Further, there is a need for messaging intended for providers that aims to foster positive perceptions about educating their patients about air pollution and exposure reducing strategies. This kind of messaging would need to address the concerns and barriers raised during the two providers discussions, including what we know and do not know about absolute risk reduction (or increase) relative to the cost of action to both the patient or to the doctor (for example using up precious time they have with the patient or using up the patient’s cognitive and financial budgets). Compared to patient-centric messaging that, for example, encourages using an air purifier, provider-centric messaging might encourage the inclusion of the topic during patient communication in the clinic as well as during cardiac rehabilitation initial intake, one-on-one conversations and group classes. Messaging repetition and reinforcement combined with positive feedback and recognition of behavior changes are critical. While initial messaging might come from a physician, message reinforcement from the broader healthcare team and potentially the patient’s social network and family support system may increase knowledge and adoption of recommended health behaviors like using an air purifier.

The Air Quality Index (AQI) [33] provides recommendations for individuals in sensitive groups, including children, older adults and people with heart or lung disease. Many AQ exposure factors are considered outside of an individual’s immediate control (for example residing close to pollution sources such as industrial facilities or roadways or in cities with high baseline levels or affected by wildfire smoke). However, in addition to societal (government, industry) control, individuals also have control over aspects of air pollution exposure and can make efforts to improve their personal exposure. Within the health messaging strategy designed for cardiac patients, content is needed that increases awareness of the problem and increases self-efficacy for adopting exposure reducing strategies, such as using an air purifier.

It is important to note the difference between risks from exposure to poor air quality and the behavioral risk factors that providers and patients are more familiar with, like smoking, high salt diets and low exercise lifestyles, which are both caused by individual behavior and addressed through modification of that behavior. Poor air quality is not caused by the individual, but exposure to that poor air quality can be mitigated through individual actions. Using an air purifier is not the same as starting an exercise routine or changing your diet. In some cases, risky behaviors are choices cardiac patients have made for decades. Using an air purifier is different in that it is not a behavior change that is replacing an existing habit. Even though it is a new behavior, using an air purifier is a low effort (although potentially economically costly) behavior that may be easier to engage in compared to starting an exercise routine or new diet. Messaging that frames using the air purifier as empowering may serve to increase patients’ self-efficacy to enact other changes.

## 5. Conclusions

Poor air quality poses a substantial health risk for patients who recently experienced a cardiovascular event. Translating this evidence into actions that may reduce the public health burden of air pollution calls for a comprehensive public health strategy which includes messaging about health risks and approaches to reduce exposures for this sensitive population. This study aims to understand factors that may influence the development and implementation of an effective public health communication strategy using an inductive approach. Findings suggest a number of important elements of successful messaging to achieve risk-reducing behavior change. By using thematic inductive analysis, we were able to identify important themes related to our key research questions about messaging around air quality health risks and exposure reducing behaviors. Through our analysis of the focus group discussions, we obtained the following key insights:

(1) Cardiac patients respond best to personalized messages, delivered in their preferred format (verbal versus written) and they are receptive to air quality messages using electronic formats. Message repetition in multiple formats supports behavior change. Doctors build up a trust relationship with their patients. This trust may present an opportunity to use doctors to establish an initial understanding of AQ as a risk factor that could be followed up through repeated messages from cardiac care team, patient networks and family support systems.
(2) Change is difficult for patients and is made more difficult when there are personal tradeoffs and costs, for example giving up going to a restaurant with family or spending money on a filter that could be spent on other activities. Cardiovascular rehabilitation staff emphasized the importance of informal patient networks and family support in achieving lasting behavior change. Social recognition of successful behavioral change and celebration can reinforce good behaviors and help to build lasting change. This may suggest opportunities to use these social networks to reinforce air quality messages or create new strategies.
(3) For patients, Information can be overwhelming and too complex to promote behavior change even when there is a desire to learn. Cardiac care teams are concerned about overwhelming patients given perceptions of the need to get patients to focus on more significant risk factors.
(4) All groups (patients, physicians and non-physician providers) in this study had low awareness of cardiovascular effects of air quality, including PM2.5.
(5) Patients are interested in air purifiers as a way to reduce exposures but want more information on whether it would be personally beneficial for them. A number of factors would influence their thinking about air purifiers, but there were not clear barriers that would be an immediate cause for rejecting use. Patients look to their cardiac care team to “prescribe” the technology.
(6) Doctors need to be convinced that (a) there is strong evidence linking AQ and cardiovascular outcomes, (b) the risk is of substantial magnitude, (c) the change in risk achieved by air purifiers (or other approaches) is substantial and d) patients will be able to apply the strategy and see real reductions in risk. Doctors see journals/meetings/conferences as the primary source for their knowledge, suggesting that providing information, training and materials using these avenues may be more effective than simply providing online training or PDFs.

Future research may use these findings to inform message content and delivery to both providers and patients that encourage information sharing about the connection between air quality and health and when and how to protect your health. 

## Figures and Tables

**Table 1:** Focus group research questions.

Group	Discussion Questions		
Patient	What is the volume, timing and content of new information a cardiac rehabilitation patient receives after their cardiovascular event and in rehab? What is that learning curve like from a patient’s perspective?	To what extent is information on air quality, associated health effects and strategies to reduce exposure currently part of that learning process?	What kinds of questions do individuals have about a high efficiency particulate air (HEPA) purifiers—its purpose, proper use, impact on their health?
Non-Physician Provider	What roles do non-physician providers fulfill in cardiovascular rehabilitation programs and beyond?	What does the patient communication system look like? Do providers know about the connection between air quality and cardiovascular health? Where do providers look for new information on a topic to be discussed with patients?	What strategies or techniques have been successful at motivating behavior change?
Physician	What does cardiovascular rehabilitation patient care look like from the physician’s perspective—timeline, key players, roles/responsibilities?	To what extent is information on air quality and cardiovascular health integrated into current care? What are some factors that may influence that integration?	What are the existing and potential windows of opportunity to incorporate new information into the communication system?

**Table 2:** Focus group findings.

Group	Summary of Findings
Patient	Patients reported a high volume of learning during cardiac rehabilitation and with virtually no emphasis or integration of air quality content. However, the patients who participated in this study were clearly open to the idea of using a device like a portable air purifier to reduce their exposure to air pollution.
Non-Physician Provider	Non-physician healthcare providers assume a variety of roles in addition to healthcare provider ranging from educator, counselor, friend and coach. The individuals in this group indicated air pollution was not a topic that is currently commonly discussed with cardiac rehabilitation patients.
Physician	The physicians who participated in this study indicated a relatively limited awareness of the recent research examining the connection between air pollution and adverse cardiovascular health outcomes. Integration of air pollution related health risk messaging into cardiac care routines. Additional education for providers on the physiological mechanisms underlying the risk, as well as health benefits of reducing exposure may facilitate integrating that information into patient interactions.
